# Dendritic Cells in the Periphery Control Antigen-Specific Natural and Induced Regulatory T Cells

**DOI:** 10.3389/fimmu.2013.00151

**Published:** 2013-06-21

**Authors:** Sayuri Yamazaki, Akimichi Morita

**Affiliations:** ^1^Department of Geriatric and Environmental Dermatology, Graduate School of Medical Sciences, Nagoya City University, Nagoya, Japan

**Keywords:** dendritic cells, subset, Foxp3, antigen, tolerance

## Abstract

Dendritic cells (DCs) are specialized antigen-presenting cells that regulate both immunity and tolerance. DCs in the periphery play a key role in expanding naturally occurring Foxp3^+^ CD25^+^ CD4^+^ regulatory T cells (Natural T-regs) and inducing Foxp3 expression (Induced T-regs) in Foxp3^−^ CD4^+^ T cells. DCs are phenotypically and functionally heterogeneous, and further classified into several subsets depending on distinct marker expression and their location. Recent findings indicate the presence of specialized DC subsets that act to expand Natural T-regs or induce Foxp3^+^ T-regs from Foxp3^−^ CD4^+^ T cells. For example, two major subsets of DCs in lymphoid organs act differentially in inducing Foxp3^+^ T-regs from Foxp3^−^ cells or expanding Natural T-regs with model-antigen delivery by anti-DC subset monoclonal antibodies *in vivo*. Furthermore, DCs expressing CD103 in the intestine induce Foxp3^+^ T-regs from Foxp3^−^ CD4^+^ T cells with endogenous TGF-β and retinoic acid. In addition, antigen-presenting DCs have a capacity to generate Foxp3^+^ T-regs in the oral cavity where many antigens and commensals exist, similar to intestine and skin. In skin and skin-draining lymph nodes, at least six DC subsets have been identified, suggesting a complex DC-T-reg network. Here, we will review the specific activity of DCs in expanding Natural T-regs and inducing Foxp3^+^ T-regs from Foxp3^−^ precursors, and further discuss the critical function of DCs in maintaining tolerance at various locations including skin and oral cavity.

## Introduction

Ralph Steinman and Zanvil Cohn discovered dendritic cells (DCs) in 1973. DCs have been shown to play a key role in the immune system to link innate and adaptive immunity (Steinman, 2012), a finding that won the Nobel Prize in 2011. DCs not only activate the immune system, but also participate in maintaining immunological-self tolerance (Steinman et al., 2003). DCs in the periphery actively induce tolerance when self-antigens are presented in the steady state (Hawiger et al., 2001; Bonifaz et al., 2002). In addition, DCs are critical antigen-presenting cells to regulate Foxp3^+^CD25^+^CD4^+^ regulatory T cells (T-regs) in the periphery (Yamazaki et al., 2006a; Yamazaki and Steinman, 2009).

T-regs are currently divided into thymic-derived Natural T-regs and peripheral Induced T-regs (Abbas et al., 2013). Recent findings have shown that Natural T-regs and Induced T-regs may be functionally and epigenetically different (Josefowicz et al., 2012; Ohkura et al., 2012; Samstein et al., 2012). The recent proposal of the nomenclature for T-regs recommends that “tT-reg (thymus-derived T-reg)” and “pT-reg (peripheral induced T-reg)” should be used instead of Natural T-regs and Induced T-regs (Abbas et al., 2013). The title of the Research Topics of Frontier Immunology is “Natural T-reg vs. Induced T-reg,” therefore, we use the term “Natural T-reg” and “Induced T-reg” in this review. The two types of T-regs appear indistinguishable on the surface, but the expression of Neuropilin can distinguish Natural T-regs and Induced T-regs in mice but not in humans (Milpied et al., 2009; Weiss et al., 2012; Yadav et al., 2012). Helios may be another marker for thymic-derived T-regs (Thornton et al., 2010), but can be expressed on Induced T-regs in some conditions (Akimova et al., 2011; Gottschalk et al., 2012). In most literature, Natural T-regs may be the mixture of thymic-derived T-regs and peripheral induced T-reg because the T-regs were purified from spleen and lymph nodes, and Helios and Neuropilin were not investigated. If CD4^+^ transgenic mice with RAG^−*/*−^ background such as OT II RAG^−*/*−^ mice are used in the literature, the T-regs should be peripheral induced T-regs because those mice lack Foxp3^+^ T-regs (Itoh et al., 1999).

In the current review, we have focused on the roles of DCs in expanding antigen-specific Natural T-regs and inducing antigen-specific Foxp3^+^ T-regs (Induced T-regs) from Foxp3^−^ CD4^+^ T cells. Recent studies indicate that DCs in different location have distinct subsets for expanding Natural T-regs and generating Induced T-regs.

## Thymic-Derived Natural T-regs are Anergic, but Can be Expanded by Antigen-Presenting DCs

Sakaguchi et al. (1995) investigated autoimmune diseases induced by neonatal thymectomy, and discovered that a subpopulation of peripheral CD4^+^ T cells that express IL-2 receptor-α (CD25) derived from the thymus play a regulatory role in maintaining immunological-self tolerance (Asano et al., 1996). Their striking finding was that CD25^+^CD4^+^ regulatory T cells exist in normal naïve CD4^+^ T cell population in the periphery with no immunization in mice. Subsequently, the groups of Shevach and Sakaguchi simultaneously reported one of the most prominent characteristics of CD25^+^CD4^+^ T-regs, specifically, “ CD25^+^CD4^+^ T-regs are anergic and suppressive upon T cell-receptor (TCR) stimulation with splenic antigen-presenting cells *in vitro*” (Takahashi et al., 1998; Thornton and Shevach, 1998). Thymic CD25^+^CD4^+^CD8^−^T cells were additionally shown to be anergic and suppressive (Itoh et al., 1999). Therefore, at that time, thymic-derived Natural T-regs were considered non-proliferative and tough to expand, which posed a major limitation, as considerable numbers of CD25^+^CD4^+^ T-regs are required to develop new treatments for autoimmunity.

Subsequently, we and others showed that CD25^+^CD4^+^ Natural T-regs can be expanded, even without exogenous IL-2, when DCs are used as antigen-presenting cells (Yamazaki et al., 2003; Fehervari and Sakaguchi, 2004). According to the recent proposal of the nomenclature for T-regs (Abbas et al., 2013), these CD25^+^CD4^+^ T-regs that we used in our experiments may be mixture of thymic-derived T-regs and peripheral induced T-reg. Both CD25^+^CD4^+^ T-regs in the periphery and thymic CD25^+^CD4^+^CD8^−^T cells produced a small amount of IL-2 following stimulation with antigen-presenting DCs (Yamazaki et al., 2003; Fehervari and Sakaguchi, 2004). Moreover, expansion of CD25^+^CD4^+^ T-regs was partially dependent on the expression of CD86 and CD80 on DCs and IL-2 (Yamazaki et al., 2003). IL-2 is important for T-reg function and survival (Malek et al., 2002; Bayer et al., 2005; Setoguchi et al., 2005). Importantly, further investigations showed that the expanded Natural T-regs by antigen-presenting DCs suppress type-1 diabetes in NOD mice (Tarbell et al., 2004) and graft-versus-host diseases (GVHD) in an antigen-specific manner (Yamazaki et al., 2006b).

## Possible Role of Monocyte-Derived DCs in Expanding Natural T-regs During Inflammation

Examination of several types of antigen-presenting cells, including resident classical DCs, for Natural T-reg expansion ability, revealed that lipopolysaccharide (LPS)-stimulated mature bone-marrow-derived DCs (BM-DCs) and lymph node DCs from complete Freund’s adjuvant (CFA)-treated mice exhibit the highest Natural T-reg expansion activity (Yamazaki et al., 2003).

Recent studies demonstrated that DC-SIGN/CD209a^+^ monocyte-derived DCs are recruited upon LPS injection, and accumulated in the T cell area of skin-draining lymph nodes (Cheong et al., 2010). DC-SIGN/CD209a^+^ monocyte-derived DCs were as functionally active as resident classical DCs when tested for capture of antigen and presentation ability to MHC class I and class II to stimulate effector T cells. Specifically, DC-SIGN/CD209a^+^ cells were recruited from blood monocytes in a toll-like receptor (TLR)-4-, CD14-, and TRIF-dependent manner (Cheong et al., 2010). CFA contains mycobacterium tuberculosis, which can stimulate TLR-4 (Kleinnijenhuis et al., 2011).

**Figure 1:**
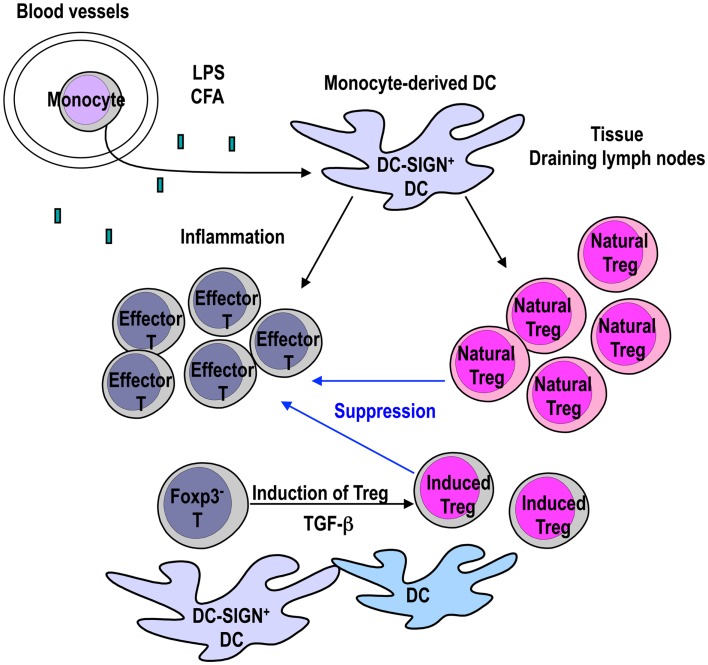
**Monocyte-derived DCs expand Foxp3^+^ T-regs to control inflammation**. DC-SIGN/CD209a^+^ monocyte-derived DCs are recruited to the site of inflammation by TLR-4 ligands such as LPS or CFA. The mature monocyte-derived DCs present antigens to not only effector T cells but also Natural T-regs. LPS-matured DCs can expand functional Natural T-regs (Yamazaki et al., 2003). If TGF-β is provided from other cells, mature DCs in the inflammatory site can stimulate the generation of Induced T-regs from Foxp3^−^ CD4^+^ T cells in the presence of antigen (Yamazaki et al., 2007). Expanded Natural T-regs and Induced T-regs may play a role in controlling inflammation.

We propose that DC-SIGN/CD209a^+^ monocyte-derived DCs expand not only effector T cells, but also Natural T-regs for the regulation of inflammation (Figure 1). This would indicate that inflammation induced by microbe signals induces DC maturation and activates effector cells, but simultaneously induces Natural T-reg expansion to control the inflammatory process (Figure 1). While inflammation is induced by several stimuli, it remains to be established whether other stimuli except TLR-4 can generate DC-SIGN/CD209a^+^ monocyte-derived DCs *in vivo*. Further studies are necessary to investigate the signals that recruit DC-SIGN/CD209a^+^ monocyte-derived DCs.

The finding that Natural T-regs can be expanded by mature DCs during inflammation is intriguing. The next issue to resolve is whether Induced T-regs are also generated during inflammation that have a role in controlling the inflammatory process. As we discussed below, Induced T-regs are generated via signaling through TGF-β LPS-matured BM-DCs are active in inducing Foxp3^+^ T-regs from Foxp3^−^ CD4^+^ T cells in the presence of active TGF-β (Yamazaki et al., 2007). TGF-β is produced as an inactive latent complex from many cells, and activation is localized to sites where TGF-β is released from latency (Li and Flavell, 2008; Yang et al., 2012). TGF-β has been shown to be activated by α_v_β_8_ integrin on the DC surface (Travis et al., 2007). Therefore, it is possible that Induced T-regs are generated by mature DCs that participate in controlling the inflammation as well as Natural T-regs, as long as TGF-β is provided from other cells (Figure 1).

## DCs are Professional Antigen-Presenting Cells that Induce Foxp3 Expression from Foxp3^−^ CD4^+^ T Cells in the Presence of TGF-β

Foxp3 is an established critical transcription factor for T-reg development and function (Fontenot et al., 2003; Hori et al., 2003; Khattri et al., 2003). In addition to thymic-derived Natural Foxp3^+^ T-regs, Foxp3^+^ T-regs are induced from Foxp3^−^ CD4^+^ T cells in the periphery, designated “Induced T-regs,” “Adaptive T-regs,” or pT-regs (Abbas et al., 2013). The finding that Foxp3^+^ T-regs are induced from Foxp3^−^CD25^−^CD4^+^ T cells with TGF-β in the periphery was initially reported by Chen et al. (2003).

To investigate whether DCs play a role in the induction of Foxp3^+^ T-regs from Foxp3^−^ CD4^+^ T precursors, using OVA CD4^+^ T-cell-receptor transgenic mice with RAG^−*/*−^ background, we compared the Foxp3 induction activities of spleen CD11c^+^DCs with DC-depleted splenocytes (Yamazaki et al., 2007). Notably, lower numbers of DCs were able to induce more Foxp3^+^ T-regs with smaller doses of peptide antigens in the presence of TGF-β (Yamazaki et al., 2007). The T-regs induced by antigen-presenting DCs were suppressive *in vitro* and *in vivo* (Yamazaki et al., 2007). DCs were also able to induce Foxp3^+^ T-reg from wild-type polyclonal Foxp3^−^CD4^+^ T precursors via allogeneic mixed leukocyte reactions. Allogeneic DCs expand functional Induced Foxp3^+^ T-regs in the presence of with TGF-β *in vitro* (unpublished data).

The collective findings indicate that DCs require only a small amount of antigen to induce Foxp3^+^ T-regs if TGF-β is provided from the environment, supporting the theory that DCs are the professional antigen-presenting cells to induce Foxp3^+^ T-regs from Foxp3^−^ precursors in the periphery.

## The Foxp3^+^ T-reg Number is Regulated by DCs *in vivo*

In keeping with the above findings that DCs expand Natural T-regs and induce T-regs from Foxp3^−^ T cells, the numbers of DCs, and Foxp3^+^ T-regs *in vivo* correlate with each other. GM-CSF, a key cytokine in DC generation (Caux et al., 1992; Inaba et al., 1992; van de Laar et al., 2012), was shown to promote Natural T-reg expansion via DC generation and prevent type 1 diabetes in NOD mice (Gaudreau et al., 2007). Similarly, repetitive injection of Fms-like tyrosine kinase 3 ligand (FLT3L), another important cytokine for DC development (Maraskovsky et al., 1996; Waskow et al., 2008), induced expansion of Natural T-regs (Swee et al., 2009). Moreover, *in vivo* ablation of Foxp3^+^ T-regs in Foxp3-diphtheria toxin (DT) receptor (Foxp3-DTR) mice led to increased DC number *in vivo* (Kim et al., 2007). Division of DC precursors is controlled by Foxp3^+^ T-regs (Liu et al., 2009), and the numbers of DCs are directly correlated with the Foxp3^+^ T-reg number *in vivo* (Darrasse-Jeze et al., 2009). Notably, Foxp3^+^ T-reg number was reduced in CD11c-DT receptor (CD11c-DTR) bone marrow chimera mice after depletion of CD11c^+^DCs (Darrasse-Jeze et al., 2009). Therefore, Foxp3^+^ T-regs and DCs appear to regulate each other *in vivo*.

However, constitutive DC-depleted mice contain normal level of Foxp3^+^ T-regs in the thymus and periphery (Birnberg et al., 2008; Ohnmacht et al., 2009). Birnberg et al. (2008) crossed CD11c-Cre BAC transgenic mice with those harboring a conditional diphtheria toxin A (DTA) transgene in the constitutively active Rosa26 locus (CD11c-DTA mice). CD11c-DTA mice constitutively lacked classical DCs, but contained spared amount of plasmacytoid DCs (pDCs) and epidermal Langerhans’ cells (LCs). These mice developed myeloproliferative diseases, but contained normal Foxp3^+^ T-reg numbers in thymus and spleen (Birnberg et al., 2008). Foxp3^+^ T-regs from CD11c-DTA mice constitutively lacking classical DCs exerted suppressive effects *in vitro* (Birnberg et al., 2008). Subsequently, Ohnmacht et al. (2009) developed similar CD11c-DTA mice with constitutive loss of all classical DCs, pDCs, and LCs. In their mice, both intrathymic Natural T-reg development and peripheral Foxp3^+^ T-reg induction were normal, but Th1 and Th17 cells were spontaneously increased (Ohnmacht et al., 2009).

Thus, there is a discrepancy in the number of Foxp3^+^ T-regs between CD11c-DT receptor (CD11c-DTR) bone marrow chimera mice (Darrasse-Jeze et al., 2009) and constitutive DC-depleted mice (Birnberg et al., 2008; Ohnmacht et al., 2009), in which may be attributed to the differences between acute and chronic DC depletion. In the case of acute DC depletion with DT, DC activity in maintaining Foxp3^+^ T-regs may be easy to detect. In contrast, upon constitutive deletion of DCs, antigen-presenting cells other than DC may be able to compensate and rescue the development and homeostasis of Foxp3^+^ T-regs in the periphery. Alternatively, in the case of Birngerb’s mice, it is possible that pDCs and LCs are sufficiently active in maintaining the Foxp3^+^ T-regs in the periphery.

Taken together, the results indicate that DCs regulate the numbers of Foxp3^+^ T-regs *in vivo*. It is possible that DCs regulate the numbers of Foxp3^+^ T-regs *in vivo* may be dependent on IL-2, which is an important cytokine both for Natural T-regs and Induced T-regs (Malek et al., 2002; Bayer et al., 2005; Setoguchi et al., 2005; Davidson et al., 2007). DCs has been shown to produce IL-2 upon LPS stimulation (Granucci et al., 2001). Treatment of anti-IL-2 antibody reduces the numbers of T-regs *in vivo* and main source of IL-2 was T cells (Setoguchi et al., 2005). However, we cannot deny the possibility that IL-2 from DCs have a role in regulating the numbers of Foxp3^+^ T-regs especially in the inflammatory condition.

The types of T-reg (Natural or Induced) regulated by DCs *in vivo* were the next focus of discussion. In FLT3-treated mice, adoptively transferred Foxp3^+^ T cells were expanded, but not converted into Foxp3^+^ T-regs (Darrasse-Jeze et al., 2009; Swee et al., 2009). Thymectomy prior to FLT-3 treatment did not affect the observed increase of Foxp3^+^ T-regs. Accordingly, it is suggested that the thymic output of Foxp3^+^ T-reg does not contribute to the increase of T-reg by FLT-3 (Swee et al., 2009). GM-CSF treatment induced expansion of Natural T-regs, but no conversion of Foxp3^−^ T cells into Foxp3^+^ T-regs *in vitro* (Zou et al., 2010). It appears that DCs expanded by FLT-3 or GM-CSF *in vivo* regulate Natural, rather than Induced T-regs.

In experiments where polyclonal Foxp3^−^CD4^+^ T cells were used for the adoptive transfer in these reports, no cognate antigen was employed (Darrasse-Jeze et al., 2009; Swee et al., 2009; Zou et al., 2010). TCR repertoires from Natural T-regs are reported to be more skewed to self antigens (Jordan et al., 2001; Hsieh et al., 2004, 2006; Sakaguchi et al., 2008). Therefore, it is possible that TCR stimulation was insufficient to induce Foxp3 in adoptive transferred Foxp3^−^ CD4^+^ T cells from wild-type polyclonal mice in these reports. Peripheral induction of Foxp3 appears to favor suboptimal TCR stimulation (Kretschmer et al., 2005; Gottschalk et al., 2010), however, TCR stimulation is required for induction of Foxp3 (Ohkura et al., 2012). Therefore, we cannot discount the possibility that the Induced Foxp3^+^ T-regs are regulated by DCs *in vivo*. For example, en earlier study by Huang et al. (2010) showed that *in vivo* injection of IL-10-treated BM-DCs promoted the conversion of Foxp3^−^CD25^−^CD4^+^ T cells into Foxp3^+^CD25^+^CD4^+^ T-regs and prevents asthma attack. In this case, IL-10-treated BM-DCs may regulate Induced Foxp3^+^ T-regs.

## Specialized DC Subsets may Expand Natural T-regs and Induced T-regs

The next issue addressed is identifying the DC subsets that regulate Foxp3^+^ T-regs *in vivo*. DCs constitute several subsets with distinct functions (Heath and Carbone, 2009; Hashimoto et al., 2011; Belz and Nutt, 2012; Steinman, 2012). Murine classical spleen DCs are divided into two major subsets, i.e., CD8^+^DEC205^+^ and CD8^−^DCIR2^+^ (Dudziak et al., 2007). Accumulating evidence indicates that antigen delivery to CD8^+^DEC205 DCs by anti-DEC205 monoclonal antibody induces Foxp3^+^ T-regs from Foxp3^−^ T cells *in vivo* (Mahnke et al., 2003; Kretschmer et al., 2005; Yamazaki et al., 2008). Moreover, Natural T-regs are expanded by CD8^−^ DCIR2^+^ DCs via model-antigen delivery by anti-DCIR2 monoclonal antibody *in vivo* (Yamazaki et al., 2008).

TGF-β plays an important role in the mechanisms of induction of Foxp3^+^ T-regs from Foxp3^−^T precursors via *in vivo* antigen targeting to DCs (Kretschmer et al., 2005). When the protein level of TGF-β production was compared, DEC205^+^CD8^+^DCs produced more TGF-β than CD8^−^DCIR2^+^ DCs in the steady state (Yamazaki et al., 2008). DEC205^+^CD8^+^DCs induced Foxp3^+^ T-regs from Foxp3^−^CD4^+^ T cells without exogenous TGF-β. The induction of Foxp3^+^ T-regs by DEC205^+^CD8^+^DCs was blocked by anti-TGF-β neutralizing antibody. Importantly, DEC205^+^CD8^+^DCs matured with poly:IC, a TLR-3 ligand, produced less TGF-β than immature DEC205^+^CD8^+^DCs DCs from steady state (Yamazaki et al., 2008). CD8^−^DCIR2^+^ DCs were more potent inducers of Foxp3^+^ T-regs after addition of TGF-β into the culture, indicating that CD8^−^DCIR2^+^ DCs can use TGF-β provided from other cells (Yamazaki et al., 2008) (Table 1).

**Table 1:** **Foxp3^+^ T-regs and classical spleen DCs in the steady state**.

	CD8^+^DEC205^+^ DCs	CD8^−^DCIR2^+^ DCs	Reference
*In vivo* OVA targeting	Induce T-regs	Expand natural T-regs	Yamazaki et al. (2008)
TGF-β production	High in steady state	Low	Yamazaki et al. (2008)
Other known feature	Cross presentation, excel in MHC I presentation	Excel in MHC II presentation	Dudziak et al. (2007), Kamphorst et al. (2010)

Recent studies in DEC205 conditional knockout mice (DEC205 Dt/DT) (Fukaya et al., 2012) support the findings that DEC205^+^CD8^+^ DCs induce Foxp3^+^ T-regs from Foxp3^−^ cells and CD8^−^ DCIR2^+^ DCs expand existing Natural T-regs (Yamazaki et al., 2008). Fukaya et al. (2012) used bone marrow chimeric mice reconstituted with bone marrow from DEC205 Dt/DT mice, lacking DEC205^+^CD8^+^DCs via DT injection. DEC205^+^DCs were depleted for about 7 days after DT injection, with a complementary increase in CD8^−^ DCs. In DEC205^+^DC-depleted mice, peripheral Foxp3^+^ T-reg numbers were increased, suggesting that existing Natural T-regs in the periphery are expanded by complementally increased CD8^−^ DCs. Upon transfer of Foxp3^−^ CD4^+^ OVA transgenic OT II T cells into the chimeric DEC205^+^DC-depleted mice, antigen-specific induction of Foxp3^+^ T-regs from Foxp3^−^ CD4^+^ T cells was impaired (Fukaya et al., 2012). Therefore, DEC205^+^CD8^+^ DCs appear critical for the induction of Foxp3^+^ T-regs from Foxp3^−^ CD4^+^ T cells in the periphery. Notably, epidermal host-derived LCs remained in chimeric DEC205^+^ DC-depleted mice, since LCs are radioresistant. Based on these results, DEC205^+^DCs other than LCs (mainly CD8^+^DEC205^+^ DCs), have greater regulatory effects on the Foxp3^+^ T-reg numbers in the periphery (Fukaya et al., 2012).

## *In vivo* Antigen-Targeting Delivery Induces Functional Foxp3^+^ T-regs in Transgenic TCR

Regarding the suppressive function of Foxp3^+^ T-regs induced by antigen targeting to DCs *in vivo*, accumulating evidence has shown that antigen delivery to the DC subset suppresses experimental autoimmune encephalomyelitis (EAE) (Hawiger et al., 2004; Stern et al., 2010; Loschko et al., 2011; Idoyaga et al., 2013). Antigen delivery to not only DEC205^+^CD8^+^ DCs but also Langerin^+^DCs and pDCs led to induction of Foxp3^+^ T regs and suppression of EAE (Loschko et al., 2011; Idoyaga et al., 2013). Some DEC205^+^CD8^+^ DCs express Langerin, and therefore, the targeting antigen to Langerin^+^ DCs may represent targeting to DEC205^+^CD8^+^ DCs. While induction of T-regs from Foxp3^−^ cells is reported as the main mechanism to suppress the autoimmunity via DC antigen delivery in these reports, it should be noted that in an *in vivo* protection assay of EAE, mice require injection with adjuvant CFA, which could expand Natural T-regs, as described above.

Another notable point is that these *in vivo* antigen delivery studies were mainly performed using transgenic mice, and therefore, the results may be artificial or specific to the experimental mice employed. In future immune therapy for autoimmunity, transplantation tolerance, and allergy, antigen targeting to DCs *in vivo* should be undertaken using polyclonal T cell repertoires. In this regard, it is very intriguing that continuous infusion of HY male peptide induced antigen-specific Foxp3^+^ T-regs in the wild-type naïve repertoire to suppress the male-graft rejection response (Verginis et al., 2008). Although subcutaneous infusion of peptide antigen by osmotic pumps was performed (Verginis et al., 2008) and not targeting antigen to DCs, it is possible that DEC205^+^CD8^+^ DCs pick up the infused peptide and present antigen to T cells. Moreover, it is important to ascertain whether antigen targeting to DCs with the polyclonal T cell repertoire in humans induces Foxp3^+^ T-regs. Targeting antigen to DCs via ASGPR in human induced IL-10 producing T-regs (Li et al., 2012). Further research on antigen delivery to DCs *in vivo* with a naïve repertoire in mice and humans is required.

## CD103^+^DCs are Specialized DCs that Induce Foxp3^+^ T-regs in the Intestine

It is well established that specialized DC subsets induce Foxp3^+^ T-regs in the intestine. The groups of Belkaid and Powrie found that gut DCs expressing CD103, the αEβ7 integrin, induce Foxp3^+^ T-regs using endogenous retinoic acid (RA) and TGF-β (Coombes et al., 2007; Sun et al., 2007). RA is known to imprint T cells expressing CD103 with gut-homing instructions (Iwata et al., 2004). Furthermore, RA, a vitamin A metabolite, controls the Th17 and Foxp3^+^ T-reg balance (Mucida et al., 2007). Importantly, CD103^+^DCs has retinoic acid dehydrogenase (RALDH) that activates RA (Coombes et al., 2007; Sun et al., 2007). RA suppresses the production of inflammatory cytokines and acts as a co-factor for TGF-β to induce Foxp3^+^ T-regs (Hill et al., 2008).

Interestingly, CD103^+^DCs are migratory DCs that carry antigens from the intestine to lymph nodes (Jaensson et al., 2008). Therefore, intestine-derived CD103^+^DCs present feeding antigens to induce Foxp3^+^ T-regs and play a key role in maintaining oral tolerance. The group of Honda showed that Foxp3^+^ T-regs in colon are possibly induced by commensal bacteria, *Clostridium* (Atarashi et al., 2011). Germ-free mice and antibiotics-treated mice had decreased numbers of Foxp3^+^ T-regs in colon, but increased or unchanged numbers of T-regs in small intestine. *Clostridium-*colonized gnotobiotic mice exhibited a robust accumulation of Foxp3^+^ T-regs in colon, but not in small intestine. Colonic epithelial cells stimulated with *Clostridum* produced TGF-β (Atarashi et al., 2011). Therefore, Foxp3^+^ T-regs are induced by *Clostridium* directly in the colon, but not in the small intestine. It is possible that Foxp3^+^ T-reg induction in the small intestine and colon may be regulated via different mechanisms.

## The Role of Vitamin D in Induction of Foxp3^+^ T-regs by DC Subsets

Another intriguing question to solve was whether vitamin D_3_ generated skin facilitates Foxp3^+^ T-reg induction, in view of the finding that RA, the active metabolite of vitamin A, play a role in inducing Foxp3^+^ T-regs in the intestine. Vitamin D_3_ (cholecalciferol) is generated in the skin in response to sun exposure, and converted to active 1,25 (OH)_2_D_3_ through an enzymatic cascade in the liver and kidney. Vitamin D controls not only calcium homeostasis but also the immune functions (Sigmundsdottir and Butcher, 2008; Baeke et al., 2010; Maruotti and Cantatore, 2010).

Active 1,25 (OH)_2_D_3_ production is controlled by 25-hydroxyvitamin D_3_-1α-hydroxylase (25(OH)D_3_-1α-hydroxylase), the mitochondrial cytochrome P450 enzyme that catalyzes the conversion of 25(OH)D_3_. Synthesis of 1,25 (OH)_2_D_3_ normally occurs in the kidney. However, it is reported that DCs participate in local production of active 1,25 (OH)_2_D_3_ in an autocrine or paracrine manner (Fritsche et al., 2003). In experiments using human monocyte-derived DCs, they found that LPS-matured DCs actively produced 1,25 (OH)_2_D_3_.

Sigmundsdottir et al. (2007) reported that the sun-light induced precursor, vitamin D_3_, is metabolized by DCs and presented as 1,25 (OH)_2_D_3_ to responding T cells. The vitamin, 1,25 (OH)_2_D_3_, induced T-cell expression of CCR10 and T-cell migration to the chemokine, CCL27. Human DCs express both 25- and 1-hydroxylase activities (CYP27A1 and CYP27B1). The group demonstrated that monocyte-derived DCs and skin-draining lymph node DCs have the capacity to activate vitamin D_3_. However, the type of DC subset activating vitamin D_3_ remains unknown and should be an exciting focus of future research. Mouse T cells upregulate CCR10 and move by chemotaxis to CCL27 less efficiently than human T cells after treatment with 1,25 (OH)_2_D_3_ (Sigmundsdottir et al., 2007; Sigmundsdottir and Butcher, 2008). Therefore, identification of the vitamin D-metabolizing DC subset that plays a role in inducing Foxp3^+^ T-regs or expanding Natural T-regs using mice may be difficult.

Although the DC subsets metabolizing vitamin D_3_ have not been determined, vitamin D clearly play a role in Foxp3^+^ T-regs (Ghoreishi et al., 2009; Jeffery et al., 2009; Urry et al., 2012). For example, Jeffery et al. (2009) reported that human CD25^−^ CD4^+^ T cells are converted into CTLA-4^+^Foxp3^+^ T-regs in the presence of mature DCs plus inactive 25(OH)D_3_. Based on this finding, it is proposed that mature DCs produce active 1,25 (OH)_2_D_3_ and induce Foxp3^+^ T-regs from Foxp3^−^ cells. Urry et al. (2012) showed that human and murine Natural T-regs maintain Foxp3 expression and are expanded in the presence of active 1,25 (OH)_2_D_3_, even with the absence of DCs in the culture. Therefore, it is possible that vitamin D play a role in the stimulation of both Natural T-regs and Induced T-regs.

## Skin DC Subsets and Foxp3^+^ T-regs

**Figure 2:**
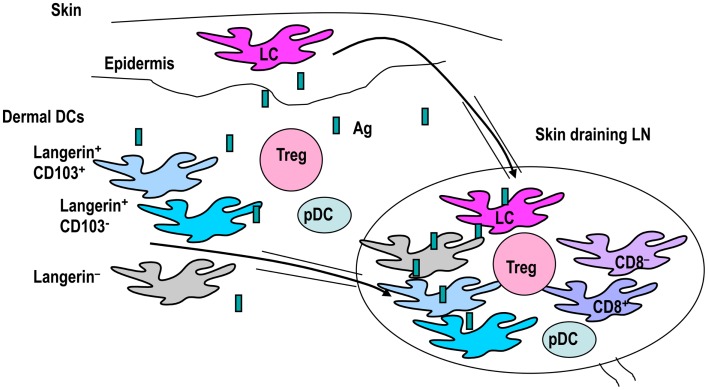
**The classification of skin DC subsets**. Skin DCs are epidermal Langerhans cells (LCs) and dermal DCs. Dermal DCs constitute Langerin^+^ and Langerin^−^ subtypes. Langerin^+^ dermal DCs are further subdivided into CD103^+^ and CD103^−^ groups. These skin DCs carry antigens and migrate to draining lymph nodes. Skin-draining lymph nodes contain many DC subsets, including skin migratory DCs, resident classical CD8^+^DCs, CD8^−^ DCs, and pDCs. Therefore, interactions between DC subsets and T-regs in skin and skin-draining lymph nodes may be complex.

Dendritic cell subsets are more complex in skin and skin-draining LNs (Figure 2). Skin-resident DCs include epidermal Langerhans cells (LCs) and dermal DCs. Langerin (CD207) is a C-type lectin mainly expressed on mainly LCs. It was originally assumed that Langerin^+^ dermal DCs constitute transit LCs from the epidermis to skin-draining lymph nodes, until a distinct population was identified (Bursch et al., 2007; Ginhoux et al., 2007; Poulin et al., 2007; Nagao et al., 2012). LCs are radioresistant and renew via *in situ* proliferation in the steady state (Merad et al., 2002; Poulin et al., 2007). Some precursors of LCs are recruited from monocytes to hair follicles after stress (Nagao et al., 2012). In contrast, Langerin^+^ and Langerin^−^ dermal DCs are constantly maintained by blood-borne radiosensitive bone marrow precursors (Bursch et al., 2007; Ginhoux et al., 2007; Poulin et al., 2007; Liu et al., 2009). Furthermore, recent reports indicate that dermal DCs are divided into three subsets: Langerin^+^ CD11b^*low*^, Langerin^−^ CD11b^−^, and Langerin^−^ CD11b^+^ (Guilliams et al., 2010b; Henri et al., 2010). In these studies, half of the Langerin^+^ CD11b^*low*^ dermal DCs appeared to express CD103, while the other half were devoid of CD103. Skin DCs constantly migrate to skin-draining lymph nodes and constitute migratory DC subsets. Therefore, in skin-draining lymph nodes, there are resident classical CD8^+^ DCs, CD8^−^ DCs, pDCs, and migratory skin DCs, including LCs and three to four dermal DCs.

Accumulating evidence has shown that lymphoid tissue-resident CD8^+^ DCs and CD103^+^ DCs are members of the same subset (Ginhoux et al., 2009; Edelson et al., 2010). CD8^+^ DCs and CD103^+^ DCs play specialized roles for cross-presentation (Bedoui et al., 2009), and their development is regulated by the same four transcription factors (Belz and Nutt, 2012). Lagnerin^+^CD103^+^ DCs are specialized to cross-present antigens (Henri et al., 2010). Moreover, CD103^+^ DCs are considered migratory DCs (Huang et al., 2010). Some resident CD8^+^DCs are known to express Langerin (Cheong et al., 2007). Malissen and colleagues proposed that the universal classification of DCs into five major subsets irrespective of tissues and species, specifically, monocyte-derived inflammatory DCs, LCs, pDCs, CD11b-type DCs, and CD8-type DCs (Guilliams et al., 2010b). Among these, CD11b-type DCs are heterogeneous (Ginhoux et al., 2009). Notably, the gut CD103^+^DCs that induce Foxp3^+^ T-regs may be different from CD8-type DCs such as dermal Langerin^+^CD103^+^DCs because the gut CD103^+^DCs express CD11b, whereas Langerin^+^CD103^+^DCs do not (Heath and Carbone, 2009). It is possible that the gut CD103^+^ DC subset is one of CD11b-type DCs (Heath and Carbone, 2009).

According to this classification, migratory CD103^+^DCs and resident CD8^+^DCs are Langerin^±^ and considered part of the same subset as CD8-type DCs. This theory is attractive since both migratory CD103^+^DCs and resident CD8^+^DCs are probably active in inducing Foxp3^+^ T-regs (Yamazaki et al., 2008; Idoyaga et al., 2013). Thus, another possible common feature of CD8-type DC may be Foxp3^+^ T-reg induction activity.

In view of the complex skin DC network, cross-talk between skin DC subsets and Foxp3^+^ T-regs may be also complex (Figure 2). Based on the universal classification system, CD103^+^ Langerin^+^ CD11b^*low*^ dermal DCs are apparently CD8-type DCs, while CD103^−^ Langerin^+^ CD11b^*low*^ dermal DCs and Langerin^−^ DCs should be CD11b-type DCs. In brief, skin CD103^+^ dermal DC subsets are CD8-type DCs. Considering that intestine CD103^+^ DCs induce Foxp3^+^ T-regs using RA, the issue of whether skin CD103^+^ DCs also activate RA and induce Foxp3^+^ T-regs was investigated by a couple of groups. Among the skin DC subsets investigated for ability to activate RA, dermal-derived CD103^−^ DCs, and not CD103^+^ DCs, produced RA and induce Foxp3^+^ T-regs in the skin-draining LNs (Guilliams et al., 2010a). Similarly, migratory CD11c^+^MHC classII^*high*^ DCs, which should contain both CD103^+^ and CD103^−^ DCs, induced Foxp3^+^ T-regs from Foxp3^−^ cells using RALDH (Vitali et al., 2012). Moreover, Langerin^+^ dermal DCs, containing CD103^+^ and CD103^−^ DCs (Henri et al., 2010), induced Foxp3^+^ T-regs in the OVA-expressing keratinocyte system (Azukizawa et al., 2011). Thus, it appears that migratory dermal DCs including both CD103^+^ and CD103^−^ DC population play a role in inducing Foxp3^+^ T-regs from Foxp3^−^ T cells in skin (Table 2).

**Table 2:** **Skin DC subsets and probable mechanisms to induce Foxp3^+^ T-regs**.

Skin DC subsets		Reference
LCs	TGF-β production	Kaplan et al. (2007), Bobr et al. (2012)
Langerin^−^CD103^−^CD11b^+^ dermal DCs	RALDH^+^	Guilliams et al. (2010a)
MHC class II^*high*^ CD11c^+^ migratory DCs (CD103^±^)	RALDH^+^	Vitali et al. (2012)
Langerin^+^ dermal DCs (CD103^±^)	TGF-β production	Azukizawa et al. (2011)

The next issue to resolve was the role of LCs in inducing Foxp3^+^ T-regs. LCs produce TGF-β (Kaplan et al., 2007; Bobr et al., 2012), and are suggested to function in maintaining tolerance rather than inducing immunity (Kaplan et al., 2005; Obhrai et al., 2008; Igyarto et al., 2009; Bobr et al., 2010; Fukunaga et al., 2010; Yoshiki et al., 2010; Kautz-Neu et al., 2011; Shklovskaya et al., 2011). Epidermal RANKL-stimulated LCs expand T-regs in transgenic mice with RANKL expression under the K14 promoter (Loser et al., 2006). Importantly, a recent study reported proliferation of human skin Foxp3^+^ T-regs with autologous LCs in the culture (Seneschal et al., 2012). However, in this report, it was unclear whether T-regs were expanded or induced from Foxp3^−^ T cells, since whole T cells were used as the starting population. It is a considerable challenge to distinguish between Natural T-reg expansion and Induced T-reg induction, because Foxp3 is easily up-regulated in humans (Walker et al., 2003; Tran et al., 2007), and it is impossible to use the Foxp3-reporter as in mice. Another recent investigation showed that LCs protect against allergic contact dermatitis by toleralizing CD8^+^ T cells through Foxp3^+^ T-regs (Gomez de Aguero et al., 2012). In this study, the conversion of Foxp3^+^ T-regs from Foxp3^−^ cells did not occur, suggesting that LCs expand already existing Natural T-regs.

Finally, Langerin^+^DC-depleted mice lacking LCs and Langrin^+^ dermal DCs did not develop autoimmune diseases (Bennett et al., 2005; Kaplan et al., 2005; Kissenpfennig et al., 2005), suggesting that other types of DCs compensate to maintain the Foxp3^+^ T-reg population.

## Possible Role of TLR-2 Signaling in Inducing Foxp3^+^ T-regs in Skin

As discussed above, skin DCs appear to play a key role in expanding Natural T-regs and inducing T-regs. It is speculated that Foxp3^+^ T-regs also function in maintaining the tolerance versus immunity in the skin (Dudda et al., 2008; Tomura et al., 2010; Naik et al., 2012). Indeed, Foxp3^+^ T-regs are enriched in skin, compared to lymphoid tissue (Sather et al., 2007; Naik et al., 2012). It would therefore be interesting to determine the type of signals controlling Foxp3^+^ T-regs in skin. One possibility is TLR signals, since skin is exposed to many commensals, among which yeast, *Staphylococcus* and *Mycoplasma* provide a source of TLR-2 ligands (Yamazaki et al., 2011). TLR-2 signaling is known to activate Foxp3^+^ T-reg induction (Chen et al., 2009; Manicassamy et al., 2009; Round and Mazmanian, 2010). Zymosan from yeast can bind to TLR-2 and dectin-1, a C-type lectin, expressed on DCs, and induces TGF-β (Dillon et al., 2006). Zymosan induces RALDH expression in DCs via a mechanism largely dependent on TLR2-mediated activation, which induces Foxp3^+^ T-regs (Manicassamy et al., 2009). Pam2 lipopeptides, derived from *Staphylococcus aureus* or *Mycoplasma*, are TLR-2 ligands (Yamazaki et al., 2011). Therefore, stimulation from yeast, *Staphylococcus* or *Mycoplasma* may be a trigger for the induction of Foxp3^+^ T-regs through TLR-2.

TLR-2 signal induces Foxp3^+^ T-regs via IL-10 production and suppresses anti-tumor response to melanoma in mice (Yamazaki et al., 2011). Other studies have consistently reported that DCs activated by TLR-2 express RALDH and induce Foxp3 (Manicassamy et al., 2009). It is unclear whether TLR-2 signals stimulate induction of Foxp3^+^ T-regs from Foxp3^*-*^ precursor cells or expansion of Natural T-regs, since most investigators used whole CD4^+^ T cells as starting population. TLR-2 induces RALHD in DCs. Therefore, it is speculated that TLR-2 signals induce Foxp3^+^ T-regs from Foxp3^−^ precursor cells as CD103^+^DCs from the intestine.

TLR-2 stimulation through commensals may additionally be required for Foxp3 induction, especially in the skin where yeast, *Staphylococcus*, or *Mycoplasma* always exist. However, a significant finding by the group of Belkaid is that the frequency of Foxp3^+^ T-regs in skin is more increased in germ-free mice (Naik et al., 2012). In cases where a skin commensal bacteria, *S. epidermidis*, was applied on the skin of germ-free mice, Foxp3^+^ T-regs were reduced and IFN-γ producing effector T cells increased in skin (Naik et al., 2012). The resident commensals in skin appear to modulate the induction of effector T cells in a Myd88/IL-1-dependent manner (Naik et al., 2012). Moreover, keratinocytes from germ-free mice actively produce IL-1 receptor antagonist (IL-1ra), leading to the suppression of effector T-cell development, and DC populations are not affected in skin (Naik et al., 2012). Thus, it appears that Foxp3^+^ T-reg and effector T cell balance is controlled directly by commensals in skin. Surprisingly, only a single type of commensal is sufficient to induce effector T cells and conversely reduce Foxp3^+^ T-regs in skin. Further studies are required to clarify whether TLR-2 signaling is required for maintaining Foxp3^+^ T-regs in skin.

## DCs Have a Role in Inducing or Expanding Foxp3^+^ T-regs in the Oral Cavity

We recently focused on the oral-cavity located between the skin and intestine, which also contains many commensals. The oral cavity is often affected by systemic immunological disorders, such as Stevens–Johnson syndrome and Behçet disease, and exposed to several antigens, including foods and pathogens, and mechanical signals via biting. Antigen administration through the oral cavity, such as sublingual (s.l.) immunotherapy, is employed to treat respiratory allergy and allergic rhinitis and conjunctivitis (Moingeon and Mascarell, 2012; Passalacqua et al., 2013). However, the mechanisms by which tolerance versus immunity are regulated in the oral cavity are unclear at present.

**Figure 3:**
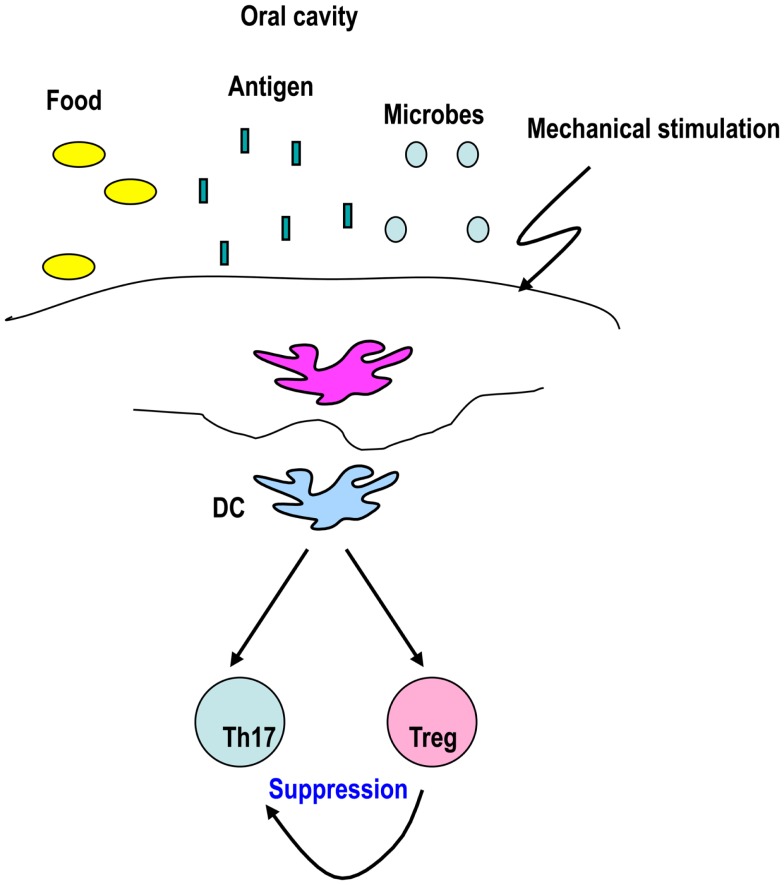
**DCs from the oral-cavity induce Foxp3^+^ T-regs to maintain tolerance**. The oral cavity is exposed to several antigens and stimuli, including foods, microbes, inhaled antigens, and mechanical stimulation. These stimuli may be sensed by DCs that play a role in generating Foxp3^+^ T-regs to maintain tolerance in the oral cavity.

Dendritic cells from the oral cavity are capable of generating Foxp3^+^ T-regs, which may maintain tolerance (Figure 3) (Yamazaki et al., 2012). CD11c^+^ DCs from oral-cavity-draining lymph nodes have the capacity to generate Foxp3^+^ T-regs in the presence of antigen *in vitro* (Yamazaki et al., 2012). It is possible that specialized DC subsets are required to induce Foxp3^+^ T-regs in the oral cavity, as CD103^+^DCs induce Foxp3^+^ T-regs in the intestine. No increase in migratory class II^*high*^ DCs, CD103^+^ DCs, CD8^+^ DCs, and pDCs in the oral-cavity-draining lymph nodes was observed (Yamazaki et al., 2012). The frequency of CD8^−^ DCs in oral-cavity-draining lymph nodes was slightly higher than in axillary lymph nodes (Yamazaki et al., 2012). In view of our former finding that 33D1^+^ CD8^−^ DCs expand Natural T-regs (Dudziak et al., 2007), it is possible that CD8^−^ DCs play a role in expanding Natural T-regs, rather than inducing Foxp3^+^ T-regs from Foxp3^−^ precursors in oral-cavity-draining lymph nodes.

Three distinct subsets of migrating DCs from the oral mucosa have been identified in the regional lymph nodes (Chalermsarp and Azuma, 2009), specifically, CD11c^*high*^ Langerin^−^, CD11c^*inter/low*^Langerin^−^, and CD11c^*inter/low*^Langerin^+^, which are all CD8 negative. It appears that CD11c^+^CD8^−^ DCs capture s.l. administered antigen and ferries it into the draining lymph nodes where both migratory CD8^−^ DCs and resident CD8^+^DCs prime the CD4 response (Song et al., 2009). S.l.-administered protein antigens are captured by DCs and are rapidly recruited to draining lymph nodes within 12–24 h, and the regulatory mechanisms established within 2–5 days in draining lymph nodes (Passalacqua et al., 2013). Both Foxp3^+^ T-regs and IL-10 producing Tr1 type T-regs are induced upon s.l. immunization (Bohle et al., 2007).

Further research is required to establish whether DCs in oral-cavity-draining lymph nodes induce Foxp3^+^ T-regs from Foxp3^−^ precursors or expand existing Foxp3^+^ T-regs. Additionally, the mechanisms underlying the maintenance of immune tolerance in the oral-cavity require clarification.

## Foxp3^+^ T-regs and DC Subsets in Humans

The majority of studies on Foxp3^+^ T-regs and DCs described above were performed using mice. It is quite important to ascertain whether the interactions of Foxp3^+^ T-regs and DCs are similar in mice and humans. As we mentioned earlier, a limited number of studies have been performed in humans showing that epidermal LCs stimulate the proliferation of Foxp3^+^ T-regs in the culture (Seneschal et al., 2012) and IL-10 producing T-reg is induced by ASGPR DCs in human (Li et al., 2012).

Murine CD8^+^DEC205^+^DCs are specialized DCs to induce Foxp3^+^ T-regs from Foxp3^−^ T cells (Yamazaki et al., 2008), but the applicability of these results to humans requires further study. Four groups simultaneously identified BDCA3(CD141)^+^ DCs as the human equivalent of mouse CD8^+^DCs (Bachem et al., 2010; Crozat et al., 2010; Jongbloed et al., 2010; Poulin et al., 2010). In terms of similarities, murine CD8^+^ DCs and human BDCA3^+^ DCs express XCR1, a chemokine receptor (Dorner et al., 2009; Bachem et al., 2010; Crozat et al., 2010, 2011; Kroczek and Henn, 2012). Moreover, both murine CD8^+^ DCs and human BDCA3^+^XCR1^+^DCs are specialized DCs for cross presentation (Dudziak et al., 2007; Dorner et al., 2009; Jongbloed et al., 2010; Poulin et al., 2010; Bachem et al., 2012). Murine CD8^+^DCs are tolerogenic DCs in mice (Hawiger et al., 2001; Bonifaz et al., 2002; Mahnke et al., 2003; Kretschmer et al., 2005; Yamazaki et al., 2008). A recent study demonstrated that BDCA3^+^DCs in skin induce IL-10 and regulatory T cells in humans (Chu et al., 2012). BDCA3^+^DCs and Foxp3^+^ T-regs are closely located within skin (Chu et al., 2012). Moreover, BDCA3^+^DCs induce CD25^*high*^ CD4^+^ T cells that suppress the alloimmune response *in vitro* and *in vivo* (Chu et al., 2012). Therefore, it is possible that BDCA3^+^DCs in humans have specialized function in inducing Foxp3^+^ T-regs, similar to CD8^+^DCs in mice.

Recent experiments showed that the function of Foxp3^+^ T-regs is recovered in psoriatic patients after exposure to ultraviolet (UV) therapy (Furuhashi et al., 2013). Interestingly, Krueger and colleagues reported increased BDCA3^+^ DCs in skin biopsy specimens after narrow band UV therapy (Kennedy Crispin et al., 2013), suggesting that BDCA3^+^DCs play a key role in Foxp3^+^ T-reg function following UV therapy. Further studies are required to examine human DC and T-reg interactions.

## Conclusion

We have discussed the roles of DCs in inducing and expanding Foxp3^+^ T-regs. Induction or expansion of antigen-specific T-regs using DCs may be provide an effective solution to treat autoimmune diseases, transplantation tolerance, and allergy. Further studies focusing on polyclonal T cell repertoires to generate antigen-specific Foxp3^+^ T-regs are warranted, particularly in humans.

## Conflict of Interest Statement

The authors declare that the research was conducted in the absence of any commercial or financial relationships that could be construed as a potential conflict of interest.
